# Chagas Disease in People with HIV: A Narrative Review

**DOI:** 10.3390/tropicalmed6040198

**Published:** 2021-11-09

**Authors:** Eva H. Clark, Caryn Bern

**Affiliations:** 1 Department of Medicine, Section of Infectious Diseases, Baylor College of Medicine, Houston, TX 77030, USA; 2 Department of Pediatrics, Section of Tropical Medicine, Baylor College of Medicine, Houston, TX 77030, USA; 3 Department of Biostatistics and Epidemiology, University of California San Francisco, San Francisco, CA 94158, USA; caryn.bern2@ucsf.edu

**Keywords:** Chagas disease, HIV, *Trypanosoma cruzi*, AIDS

## Abstract

Many questions remain unanswered regarding the epidemiology, pathophysiology, diagnosis, treatment, and monitoring of *Trypanosoma cruzi* infection in people with HIV (PWH). The reported prevalence of *T. cruzi* infection in PWH living in endemic countries ranges from 1–28% and is likely similar in at-risk US populations. While classic cardiac and gastrointestinal presentations of chronic Chagas disease occur in PWH, PWH are additionally at risk for a severe and often fatal form of *T. cruzi*-mediated disease called reactivation disease. *T. cruzi* reactivation typically occurs in PWH with low CD4 counts and poor virologic control. National HIV guidelines in several endemic South American countries recommend that all PWH be screened for *T. cruzi* infection at the time of HIV diagnosis; however, this recommendation is not widely implemented. The early detection of *T. cruzi* infection in PWH is critical as the sequelae of Chagas disease, including *T. cruzi* reactivation, may be preventable through the restoration of robust cellular immunity via the initiation of antiretroviral therapy and the appropriate use of antitrypanosomal therapy.

## 1. Introduction

Chagas disease is caused by infection with the protozoan parasite *Trypanosoma cruzi*. More than six million people are estimated to be infected in the Americas [1]. In endemic countries such as Argentina, Brazil and Bolivia, 1–32% of people with HIV (PWH) are coinfected with *T. cruzi* [2,3,4,5,6]. While co-infected PWH can develop the classic cardiac and gastrointestinal manifestations of chronic Chagas disease seen in 20–30% of their HIV-negative counterparts, they have a higher risk of morbidity and mortality because their compromised cellular immunity may permit *T. cruzi* reactivation. Reactivation typically presents as central nervous system (CNS) [7,8,9,10,11] disease and/or acute myocarditis [12]. CNS reactivation carries a high mortality rate (79–100%) if untreated [13]. Prompt recognition and antiparasitic treatment are essential to maximize the likelihood of survival [7]. In this article we present a narrative review of the literature describing Chagas disease in PWH.

## 2. Epidemiology

The prevalence of *T*. *cruzi* infection in people with HIV (PWH) generally reflects the prevalence in the at-risk HIV-negative population in the same location [14]. Reported rates of *T. cruzi*–HIV co-infection range from 1.3–5% in Brazil [5] to 1.2–4.2% in Argentina (7.1% in intravenous drug users) [4,15], and to 28% in a single Bolivian study [2,4,6,15]. In Latin American PWH living in non-endemic countries, *T. cruzi* co-infection rates range from 0% in one US screening study [16] to 1.9–10.5% in Spain [17,18,19]. Reactivation disease is thought to occur in a large minority (15–35%) of co-infected PWH who are not being treated with antiretroviral therapy (ART) [14,20,21]. Published data suggest that births to HIV-*T. cruzi* co-infected women are more likely to result in congenital Chagas disease than those who are born to immunocompetent *T. cruzi-*infected women (>75% vs. 1–5%) [22,23,24].

## 3. Pathophysiology

In both acute and chronic *T. cruzi* infection, CD4+ T helper 1 (Th1) cells are thought to be primarily responsible for the induction of protective immunity [25]. Thus, it makes sense that co-infected PWH, particularly those with low CD4 counts, have higher parasitemia levels than their HIV-negative counterparts, even in the absence of symptomatic reactivation disease [26]. The regulation of *T. cruzi* infection likely depends on the host’s balance of Th1 cells (which produce cytokines such as interferon (INF)-gamma and interleukin (IL)-2) and Th2 cells (which produce cytokines such as IL-4, IL-10, and tumor growth factor (TGF)-beta) [25,27]. Though few data describing the interaction between HIV and *T. cruzi* are available, murine models support the link between the suppression of T cell activity and the inability to control *T. cruzi* parasitemia [28]. Silva et al. demonstrated increased IL-10 levels in an immunosuppressed murine model of *T. cruzi* coinfection with murine leukemia virus [29], and other murine models indicate that monoclonal antibody-mediated neutralization of IL-10 and TGF-beta reverses suppressed IFN-gamma responses and susceptibility to parasitic disease [30]. While these data partially explain the susceptibility of PWH with low CD4 counts to *T. cruzi* reactivation, the immunologic effector mechanisms that trigger and permit reactivation remain unclear.

Regarding the pathologic characteristics of the parasite itself, though polyclonal *T. cruzi* infections occur in PWH with both chronic and reactivated *T. cruzi* infection [31], immunosuppression does not appear to affect *T. cruzi* genetic diversity and population structure, and no particular *T. cruzi* discrete typing unit (DTU) has been shown to be more likely to cause reactivation disease in PWH [32]. Interestingly, Andreani et al. demonstrated that *T. cruzi* inhibits HIV replication at several stages in the macrophages, but the clinical significance of this observation is unclear [33]. Further studies are needed to better understand the pathophysiology of HIV and *T. cruzi* co-infection.

## 4. Presentations

Most PWH with Chagas disease acquired *T. cruzi* via exposure to the triatomine vector in their country of origin, although infection via other routes, such as intravenous drug use (IVDU), has been reported [34,35]. Parasite persistence and the effectiveness of the host immune response determine the clinical manifestations of *T. cruzi* infection in PWH. Both *T. cruzi* reactivation disease in PWH and chronic co-infection without reactivation are well-described in the published literature.

*Chronic Chagas disease, cardiac and gastrointestinal manifestations:* Whether PWH are more susceptible to cardiomyopathy when co-infected with *T. cruzi* remains unclear [26]. One Brazilian group investigated potential associations between *T. cruzi* parasitemia and the impact of HIV infection on cardiomyopathy [36]. Their data suggest that HIV infection may protect against the development and progression of cardiopathy, potentially due to a synergistic effect of HIV and/or ART or reduced cellular immunity attenuating a type-1 helper T-cell-mediated response in the myocardium. In a single study in Brazil, PWH were more likely to be diagnosed with digestive Chagas disease compared to their HIV-negative counterparts [26].

*Reactivation disease:* The definitions used for reactivation vary across the published literature. In the setting of HIV–*T. cruzi* coinfection, the most widely used criteria require clinical manifestations that are not typical of chronic Chagas disease plus the demonstration of the parasite by microscopy in blood, cerebrospinal fluid [CSF], or other fluids or tissue [14,37]. However, some authors also include microscopically detectable parasitemia without a reactivation-defining syndrome [6,20]. Even in the absence of positive microscopy, parasitemia levels, as defined by molecular methods, are significantly higher in asymptomatic immunosuppressed patients compared to in immunocompetent patients with chronic *T. cruzi* infection [38].

Cases of symptomatic reactivation are reported in patients with long-standing suppressed cellular immunity. A 1982 publication first reported a case of Chagas disease reactivation in a patient who was immunocompromised due to therapy for a hematological malignancy [39]. Others have described reactivation disease in patients post-solid organ transplant [40]. After the arrival of HIV to *T. cruzi*-endemic regions, Del Castillo et al. published the first recognized case of reactivated *T. cruzi* infection in a PWH in 1990 [41] (though a Brazilian group had reported *T. cruzi* antibodies in the CSF of PWH in 1988 [42] and *T. cruzi* trypomastigotes in the CSF of two PWH in 1989 [43]). Nearly all of the published cases of *T. cruzi* reactivation describe PWH naïve to ART; however, no studies evaluating risk of reactivation in PWH taking versus not taking ART exist.

Reactivation disease most frequently presents in the CNS as space-occupying cerebral lesions and/or meningoencephalitis (75–80% of cases) [7,44,45]. CNS *T. cruzi* reactivation typically manifests as nodular lesions (often called chagomas) comprising macrophages, neutrophils, microglia, astrocytes, and perivascular lymphocytes (see “Diagnosis of CNS Reactivation” section below) [9]. Astrocytes are the most abundant brain cells and are regarded as important components of the pathophysiology of CNS Chagas disease. Exposure to reactive oxygen species, which are promoted by HIV infection, seems to facilitate *T. cruzi* infection of and multiplication in astrocytes [46]. CNS reactivation is the most rapidly lethal presentation of Chagas disease in PWH. It is more common in PWH with low CD4 cell counts [44] (particularly less than 100 cells/mm^3^) and is considered to be an opportunistic infection in people with AIDS. IL-18 serum concentrations may influence or predict the risk of *T. cruzi* reactivation in PWH [36].

Reactivation manifesting as cardiac involvement (typically acute myocarditis) also occurs (10–55% of cases) [7,14]. Distinguishing between cardiac disease caused by *T. cruzi* reactivation versus the progression of pre-existing cardiomyopathy can be challenging but is important, as treatment recommendations differ [47]. Less commonly, reactivation may manifest as cervicitis [48], gastrointestinal disease [49,50], peritonitis [51], or skin lesions (specifically, erythema nodosum) [26,52]. The mortality rate of symptomatic *T. cruzi* reactivation disease is high (>75%) [20,44,45], which, in part, is due to late recognition by health care providers.

## 5. Screening and Diagnosis

*Screening for chronic Chagas disease in PWH:* HIV guidelines in several South American countries where Chagas disease is endemic, such as in Argentina and Brazil, recommend that all PWH be screened for *T. cruzi* infection at the time of HIV diagnosis or entry to care through the use of sensitive *T. cruzi* serologic testing [53]. However, limited resources often impede the large-scale implementation of this recommendation. Similarly, US and Spanish HIV guidelines recommend that all PWH with Chagas disease risk factors be screened [13,54,55]. Despite this, only a minority of at-risk PWH are screened appropriately [56].

*Diagnosis of chronic Chagas disease in PWH:* Current recommendations for initial diagnostic testing for chronic Chagas disease among PWH are the same as for the general population, i.e., confirmed diagnosis requires positive results by two or more serologic tests based on different antigens or techniques [57]. Importantly, *T. cruzi* serology may be negative in co-infected PWH with significant immunocompromise; negative serology in the setting of a high index of suspicion should prompt further testing [34,44]. Even asymptomatic PWH with chronic Chagas disease have significantly higher levels of *T. cruzi* parasitemia than HIV-negative people with chronic *T. cruzi* infection [26,32]. The level of parasitemia in PWH with chronic Chagas disease generally shows an inverse correlation with CD4 counts [6,32]. PCR sensitivity in chronic *T. cruzi* infection is variable but is generally higher in the coinfection setting because of the higher parasite load distribution [6,58,59].

*Diagnosis of T. cruzi reactivation disease in PWH*: The early diagnosis of reactivated *T. cruzi* infection is critical. The differential diagnosis of meningoencephalitis and the cerebral space occupying the lesions (and other common presentations of reactivation disease, such as myocarditis) in PWH is broad, and direct microscopy is often negative in the setting of CNS reactivation [14]. As mentioned previously, *T. cruzi* serologic tests can be negative in severely immunocompromised PWH. Traditional microscopic diagnosis is inexpensive but is also highly operator dependent and has relatively low sensitivity [13,35]. Thus, the possibility of *T. cruzi* infection cannot be absolutely ruled out by negative serology and microscopy, especially when reactivation disease is suspected [34,44,45,60]. In such cases, other body fluids and/or tissues should be examined by microscopy and PCR guided by clinical findings and imaging [13,61]. A high peripheral parasite load determined by molecular methods may also be used as supporting evidence of reactivation in the setting of a typical CNS or cardiac picture [62].

In transplant recipients with pre-existing chronic Chagas disease, recommendations include routine monitoring with quantitative molecular methods as well as testing during febrile episodes; the finding of rising parasite loads in serial specimens is accepted as a sufficient indicator of reactivation necessitating antitrypanosomal therapy [63,64]. Similar monitoring has never been evaluated in HIV–*T. cruzi*-coinfected patients. Although investigators have speculated about the utility of the pre-emptive antitrypanosomal treatment of asymptomatic HIV–*T. cruzi*-coinfected patients based on high peripheral blood parasite load, there is no consensus on the treatment criteria in the absence of symptomatic reactivation [6,52]. When effective ART is instituted in coinfected patients, parasite loads tend to fall rapidly in the absence of antitrypanosomal therapy, making prospective monitoring much less useful for PWH than it is transplant recipients.

*New developments in T. cruzi infection diagnosis in PWH:* One new molecular assay with many of the advantages of PCR but without the requirement of expensive equipment is called the loop-mediated isothermal amplification method (LAMP). Argentine investigators recently developed such a test to detect *T. cruzi* genetic material, the *T. cruzi* Loopamp kit (Tc LAMP) [65]. Compared to standardized quantitative real-time PCR (qPCR), Tc LAMP sensitivity and specificity were 93% (95% CI: 77–99) and 100% (95% CI: 80–100), respectively, and the agreement between Tc LAMP and qPCR was very close (κ = 0.92, 95% CI: 0.62–1.00). Tc LAMP and qPCR test results were positive for all six PWH (including tests on six blood samples and one cerebrospinal fluid [CSF] sample). Another group recently developed a urine *T. cruzi* antigen test (called Chunap) [59]. Of 31 PWH with positive *T. cruzi* serologic tests, Chunap detected 100% (7/7) of PWH with reactivation, 91.7% (11/12) of PWH with moderate parasitemia, and 41.7% (5/12) of PWH with negative parasitemia. Finally, for PWH who are suspected of having reactivation disease and who are being evaluated in high-resource settings, clinicians can consider tissue sample evaluation via the sequencing of the internal transcribed spacer 2 and D2 regions of the 28S rRNA gene, particularly in PWH with an unrevealing microscopic evaluation of blood and CSF specimens [66]. The D2 primers used in 28S rRNA gene sequencing react with multiple protozoa and fungi and therefore can detect not only *T. cruzi* but also other pathogens that share the D2 subunit and are often involved in the differential diagnosis of meningoencephalitis in PWH, such as *Toxoplasma gondii*, *Cryptococcus* spp., *Histoplasma* spp., and *Leishmania* spp. The ability to identify one of many potential pathogens with a single test is advantageous for the timely institution of the appropriate treatment and improved outcomes.

*Diagnosis of CNS reactivation:* CNS reactivation in PWH is usually suspected due to presentation with neurologic symptoms in conjunction with abnormalities on head imaging in the setting of Chagas disease risk factors. Importantly, the absence of lesions on a computed tomography of the head (CTH) does not rule out CNS involvement. If available, magnetic resonance imaging (MRI) of the brain is the preferred imaging study [45]. Rim-enhancing cerebral lesions are often seen in patients with CNS reactivation, though as many as 15% of patients may have normal brain imaging by both CTH and MRI [45]. Because the differential diagnosis of rim-enhancing cerebral lesions in PWH is broad, imaging alone is insufficient to make the diagnosis of CNS *T. cruzi* reactivation. For instance, cerebral lesions due to *Toxoplasma* encephalitis are generally indistinguishable from those caused by *T. cruzi* reactivation, though some experts believe that *Toxoplasma* more often causes cortical or basal ganglia lesions, while *T. cruzi* tends to cause white matter or subcortical lesions [67]. *Toxoplasma* and *T. cruzi* CNS disease can occur simultaneously [68,69]. The visualization of trypomastigotes in the CSF provides a definitive diagnosis of CNS reactivation [20,45]. CSF fluid analyses often show low to moderate white blood cell counts (<100 per mL of CSF) that are predominantly lymphocytic with elevated protein and low glucose levels [7,45]. If less invasive techniques fail to confirm the diagnosis, then a brain biopsy may be necessary to confirm the diagnosis [7]. If the CSF is initially positive by PCR, some experts suggest serial evaluation to monitor treatment response [70].

## 6. Treatment

*T. cruzi* reactivation disease is likely preventable with timely, sustained immune reconstitution via the administration of ART to coinfected patients. Whether pre-emptive antitrypanosomal therapy further decreases reactivation risk is unclear. With no proven test of a parasitological cure, *T. cruzi*-infected PWH must be considered to remain at risk for reactivation, even after receiving a course of antitrypanosomal therapy.

If reactivation occurs, immediate antitrypanosomal therapy plus the initiation or optimization of ART has been shown to reduce mortality [20,44,45]. If relapse occurs after initial treatment for reactivation, a course of antitrypanosomal therapy should be repeated. Two antitrypanosomal drugs—benznidazole and nifurtimox—are available for the treatment of *T. cruzi* infection. Currently there are no specific guidelines for treatment regimens for co-infected PWH, though some experts recommend longer therapy derations [54].

*Benznidazole:* Oral benznidazole (5–7 mg/kg/day in 2 doses for 60–90 days) is the most used antitrypanosomal agent and is usually chosen as the first-line treatment for chronic or reactivated Chagas disease in adult patients. However, our understanding of benznidazole pharmacokinetics and pharmacodynamics is limited, particularly in PWH. Consequently, the treatment of *T. cruzi* CNS reactivation disease is guided by limited empirical knowledge. The usual benznidazole dose may be suboptimal in PWH with *T. cruzi* meningoencephalitis. Investigators measured CSF and plasma benznidazole levels in one Argentine case series of six patients, from whom they obtained 6 CSF and 19 plasma samples [71]. Only three of the six CSF samples had detectable benznidazole levels, and all were at sub-therapeutic concentrations (<2 µg/mL). Thirteen plasma samples had adequate benznidazole levels. These data suggest that higher levels of benznidazole may be necessary to adequately and/or rapidly treat CNS reactivation disease (note that a separate evaluation demonstrated that it may take more than two weeks of benznidazole treatment at the currently recommended dosing to clear *T. cruzi* from the CSF [70]). Importantly, even with currently recommended dosing regimens, adverse reactions to benznidazole therapy are common in PWH [45], just as they are in the general population; thus, close monitoring is essential. Though benznidazole is usually contraindicated in pregnancy, at least one case report demonstrates its successful use in a co-infected pregnant woman with CNS *T. cruzi* reactivation disease at 32 weeks of pregnancy [72].

*Nifurtimox:* Clinical experience with oral nifurtimox (8–10 mg/kg/day in 2 or 3 doses for 60–120 days) is even more limited than that with benznidazole [68,73]. Nifurtimox is usually less well-tolerated than benznidazole and is therefore reserved for use as a second-line therapy in patients who are unable to tolerate benznidazole.

*New/repurposed therapies under study:* Benznidazole remains the first-line drug for the treatment of all forms of Chagas disease (regardless of HIV status). However, several new and re-purposed medications are under study. In vitro studies indicate that protease inhibitors such as lopinavir and nelfinavir may have some direct activity against *T. cruzi* [74]. Reports of treatment with itraconazole [75,76,77], fluconazole, ketoconazole [76], posaconazole [78], and allopurinol [77] exist. However, posaconazole had low efficacy in randomized clinical trials in immunocompetent hosts and is considered trypanostatic rather than trypanocidal [79,80]. No rigorous efficacy data exist for the other oral antifungal agents or allopurinol.

*Starting ART and risk of immune reconstitution inflammatory syndrome:* Restoring robust cellular immunity is critical to survival in symptomatic reactivation disease, but data are lacking regarding the optimal timing of ART initiation and the choice of specific agents. Because benznidazole and nifurtimox are thought to be primarily metabolized in the liver by the cytochrome P450 system [81], it may be wise to avoid types of ART that are similarly metabolized, such as many non-nucleoside reverse transcriptase inhibitors (NNRTIs) and protease inhibitors (PIs). Integrase inhibitors (IIs), on the other hand, are not substrates of the cytochrome P450 system; thus, they likely will not have significant interactions with benznidazole or nifurtimox [82]. Though immune reconstitution inflammatory syndrome (IRIS) can theoretically occur post-ART initiation, it has not yet been documented for *T. cruzi* co-infection (except possibly in one co-infected PWH who developed erythema nodosum after starting ART [52]); thus, at this time, co-infection is not a contraindication to starting ART.

*T. cruzi secondary chemoprophylaxis in PWH and low CD4 counts:* The utility of secondary prophylaxis to prevent the reactivation of *T. cruzi* infection in co-infected PWH with low CD4 counts remains unclear. Secondary prophylaxis for PWH treated for *T. cruzi* infection is often recommended in countries with relatively high *T. cruzi* prevalence [73]. In one Argentine case series, after initial treatment for *T. cruzi* CNS reactivation disease, two PWH were successfully treated with benznidazole secondary prophylaxis at 5 mg/kg/d for three days a week until achieving CD4 counts higher than 200 cells/mm^3^ and an undetectable HIV viral load [83].

## 7. Future Directions

Many gaps remain in our knowledge of the epidemiology, pathophysiology, diagnosis, treatment, and monitoring of HIV–*T. cruzi* co-infection. A better understanding of these issues can help reduce morbidity and mortality related to co-infection in both endemic and non-endemic countries. Specific topics include (1) better defining the prevalence of and risk factors for HIV–*T. cruzi* co-infection to target interventions to those who are most at risk; (2) elucidating the immunologic effector mechanisms underlying reactivation; (3) determining whether parasite factors, such as specific genotype or polyclonality, alter the risk or location of reactivation; (4) the optimization of algorithms for *T. cruzi* screening and diagnosis, particularly in PWH with low CD4 counts; (5) placing therapeutics of coinfected patients on a more solid footing, including best practices for therapeutic drug level monitoring, identifying factors leading to low CSF drug levels, and developing better treatment regimens; (6) evaluating criteria and regimens for secondary chemoprophylaxis; and (7) ensuring that candidate test-of-cure assays are evaluated and function acceptably in coinfected PWH with significant immunosuppression.

## Data Availability

Not applicable.
